# Design and Analysis of a Neuromemristive Reservoir Computing Architecture for Biosignal Processing

**DOI:** 10.3389/fnins.2015.00502

**Published:** 2016-02-01

**Authors:** Dhireesha Kudithipudi, Qutaiba Saleh, Cory Merkel, James Thesing, Bryant Wysocki

**Affiliations:** ^1^NanoComputing Research Laboratory, Department of Computer Engineering, Rochester Institute of Technology Rochester, NY, USA; ^2^Information Directorate, Air Force Research Laboratory Rome, NY, USA

**Keywords:** neuromemristive systems, reservoir computing, memristors, process variations, epileptic seizure detection and prediction, EMG signal processing, neuromorphic hardware, neuromorphic

## Abstract

Reservoir computing (RC) is gaining traction in several signal processing domains, owing to its non-linear stateful computation, spatiotemporal encoding, and reduced training complexity over recurrent neural networks (RNNs). Previous studies have shown the effectiveness of software-based RCs for a wide spectrum of applications. A parallel body of work indicates that realizing RNN architectures using custom integrated circuits and reconfigurable hardware platforms yields significant improvements in power and latency. In this research, we propose a neuromemristive RC architecture, with doubly twisted toroidal structure, that is validated for biosignal processing applications. We exploit the device mismatch to implement the random weight distributions within the reservoir and propose mixed-signal subthreshold circuits for energy efficiency. A comprehensive analysis is performed to compare the efficiency of the neuromemristive RC architecture in both digital(reconfigurable) and subthreshold mixed-signal realizations. Both Electroencephalogram (EEG) and Electromyogram (EMG) biosignal benchmarks are used for validating the RC designs. The proposed RC architecture demonstrated an accuracy of 90 and 84% for epileptic seizure detection and EMG prosthetic finger control, respectively.

The material was assigned a clearance of CLEARED on 10 Nov 2015, Case Number: 88ABW-2015-5512.

## 1. Introduction

Neuromorphic computing has been envisioned as a highly efficient processing platform by several scientists such as Von Helmholtz (1867), Faggin, and Mead (1990). Since then several approaches toward modeling computation in biological neural systems have been demonstrated with both spiking and non-spiking neurons. The common models for computations, such as the deterministic Turing machines or attractor neural networks, do not particularly carry out computations on continuous streams of inputs. Hopfield's groundbreaking work on Recurrent Neural Networks (RNN; Hopfield, 1982) to Werbos's training the gradients of the RNN to compute with backpropagation through time (Werbos, 1990), address the complex temporal machine learning tasks. However, the challenge in using RNNs is a very unstable relationship between the parameters and the dynamics of the hidden states, referred to as the “fading or exploding gradients.” Thereby, the application of RNNs to real-world signal processing is limited. Recently, Hessian-Free optimizers (Martens, 2010) and RNNs with gated connections (Sutskever et al., 2011) are proposed to apply them to challenging sequence problems. It is important to explore models that capture the complex dynamical responses on spatiotemporal scales with heterogeneous components and easily applied to real-world problems, which are earmarks for an intelligent system. One significant advancement in this direction is the proposal to leave RNNs untrained and then later process using a simple classification/regression technique. This idea was developed in tandem by two independent research groups, Jaeger (Jaeger, 2001; Jaeger and Haas, 2004) as the Echo State Network and Maass as the Liquid State Machine (Maass et al., 2002; Buonomano and Maass, 2009). Collectively these models are referred to as reservoir computing (RC). The reservoir can also be viewed as a complex non-linear dynamic filter where the input is projected onto a high dimensional temporal map. The RC is strongly inspired by the excitatory and inhibitory networks of the cerebral cortex which are inherently unstable. Neurophysiologists have demonstrated that the excitatory networks generate further excitation (simple and predictable) and the inhibitory networks generate non-linear effects (complex). Networks that are built from both inhibitory and excitatory elements can self-organize and generate complex properties (Buzsaki, 2006). Such continuous perturbations are possible in the cortex due to the diversity of the components (neurons and synapses) and the specificity of their connections.

Several research groups are studying the theory, modeling, and software realizations of the reservoirs in real-world applications (e.g., object recognition, speech recognition, robotic movement control, dynamic pattern classification, and chaotic time-series generation). Heterogeneous software realizations of reservoirs demonstrate that the performance of these networks either equal or outperform the state-of-the-art machine learning techniques, within a given set of constraints. Though there is a growing body of research in the algorithmic study, none of the existing work explores a comprehensive hardware architecture for an energy efficient reservoir. A reconfigurable hardware architecture can significantly improve the power dissipation, area efficiency, and portability of the reservoirs. In current literature, there are hardware implementations of the reservoirs which are primarily digital (Schrauwen et al., 2007; Merkel et al., 2014). Few research groups have also explored stochastic bit-streams and mixed-signal designs for the reservoir (Schürmann et al., 2004; Verstraeten et al., 2005). However, these designs are emulating a layer of the ESN or LSM and not the overall design. Moreover, in a large scale ESN the number of synapses and neurons grow significantly and require devices such as memristors to realize these primary components in an energy efficient manner. In this research, we propose scalable and reconfigurable neuromemristive architectures for the reservoirs with specific focus on the biosignal processing applications.

Neuromemristive systems (NMSs) are brain-inspired, adaptive computer architectures based on emerging resistive memory technology (memristors).The specific memristor model used in this NMS is a semi-empirical model derived from the works of Simmons (Simmons, 1963; Simmons and Verderber, 1967), and Mott and Gurney (1940). Detailed description of the model is presented in Section 5.1. The core building blocks of an NMS are memristor based synapse, neuron, and learning circuits. The ESN architecture leverages these primitive blocks for area and power efficiency. An in-depth comparison of the proposed ESN architectures in pure digital and mixed-signal designs is validated for two different benchmarks, Electroencephalogram (EEG) and Electromyogram (EMG) biosignal datasets. Specific contributions of this research are: (i) design of a toroidal ESN architecture with hybrid topology; (ii) digital realization of the ESN architecture, ported onto different FPGA platforms; (iii) mixed-signal design of the ESN architecture with subthreshold circuits; (iv) new bipolar input synapse that leverages mismatch; (v) analyze the impact of random mismatch-based synapses on the area and power of the ESN; and (vi) performance and power analysis of the digital and mixed signal realizations of the ESN. All design abstractions of the ESN architecture are verified and tested for the EEG and EMG biosignals with applications in epileptic seizure detection and prosthetic finger control.

The rest of the paper is organized as follows: Section 2 discusses the theory and background of the reservoirs (particularly ESNs), Section 3 presents the proposed ESN architecture and the different topologies, Section 4 discusses the digital implementation of the proposed ESN architectures along with the portability to custom FPGA fabrics, Section 5 presents new mixed signal circuit building blocks for the ESN architecture and explores their design space, Section 6 discusses the biosignal benchmarks used for validation, Section 7 entails results and detailed analysis of the proposed ESN architecture, and Section 8 concludes the work.

## 2. Echo state network: theory and background

**Figure 1:**
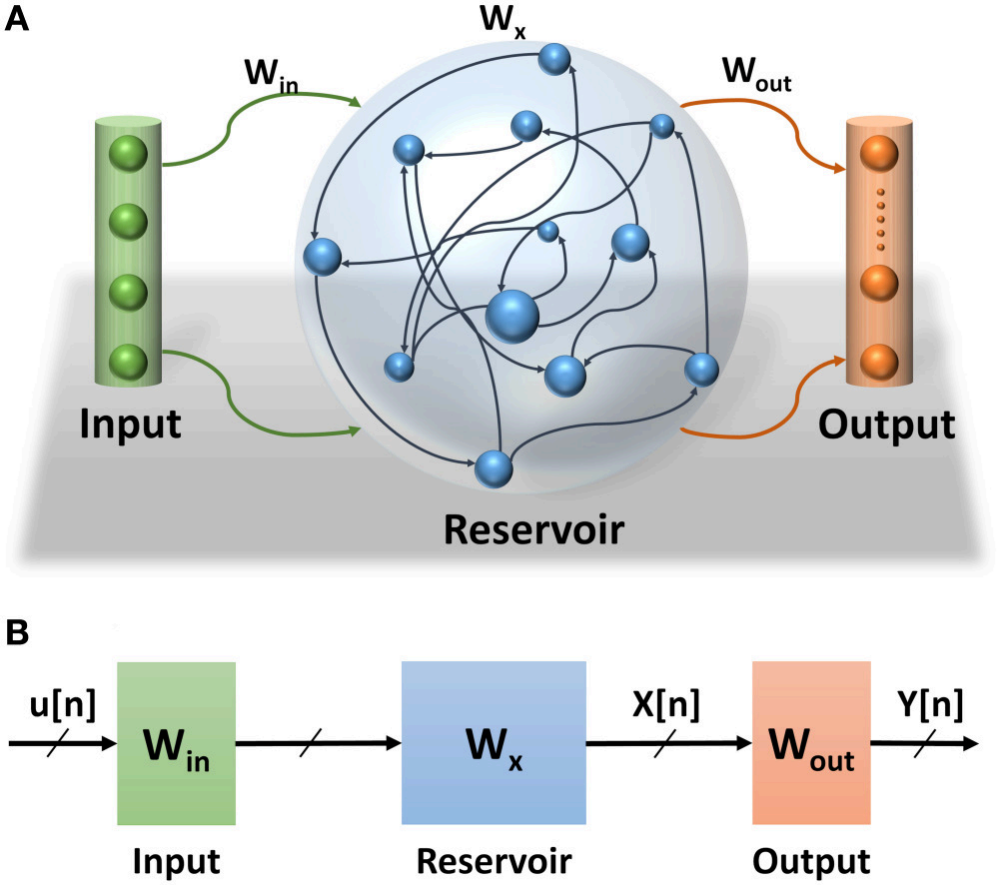
**(A)** Echo State Network consists of three layers: input layer, reservoir layer, and output layer. **(B)** Echo State Network abstract structure. How signals propagate through the ESN and the effects of different weight sets in the network.

Echo State Network (ESN) is a class of reservoir computing model presented by Jaeger (2001). ESNs are considered as partially-trained artificial neural networks (ANNs) with a recurrent network topology. They are used for spatiotemporal signal processing problems. The ESN model is inspired by the emerging dynamics of how the brain handles temporal stimuli. It consists of an input layer, a reservoir layer, and an output layer (see Figure 1A). The reservoir layer, is the heart of the network, with rich recurrent connections. These connections are randomly generated and each connection has a random weight associated with it. Once generated, these random weights are never changed during training or testing phases of the network. The output layer of the ESN linearly combines the desired output signal from the rich variety of excited reservoir layer signals. The central idea is that only the output layer weights have to be trained, using simple linear regression algorithms. ESN provides a high performance mathematical framework for solving a number of problems. Specifically, they can be applied to recurrent artificial neural networks without internal noise. ESNs have simplified training algorithms compared to other recurrent ANNs and are more efficient than kernel-based methods (e.g., Support Vector Machines) due to their ability to incorporate temporal stimuli (LukošEvičIus and Jaeger, 2009). Because of its recurrent connections, the output of the reservoir depends on the current input state and all previous input states within the system memory. The recurrent network topology of the reservoir enables feature extraction of spatiotemporal signals. This property has been used in several application domains such as motion identification (Ishu et al., 2004), natural language analysis (Tong et al., 2007), and speech recognition (Skowronski and Harris, 2007).

### 2.1. Training algorithm

Three main sets of weights are associated with the ESNs (see Figure 1B). The weights at the input and reservoir layers are randomly generated. These layers are used to extract temporal features of the input signal. They can be thought of as an internal pre-process step that prepares the signal for the actual processing layer where the classification is learned at the readout layer. Figure 1B also shows the propagation of the signals through the ESNs. The input signal to the ESN *u*(*n*) is pre-processed at the input and reservoir layers to extract the temporal feature signal *x*(*n*) which is fed to the readout layer to complete the classification process. Considering that the input and reservoir layers are not actual parts of this process, their weights are not trained, which makes training the ESNs much easier than other types of RNNs.

The goal of the training algorithm is to calculate the weights at the output layer based on the dynamic response (states) of the reservoir layer (Jaeger, 2002). The states of the reservoir layer are calculated based on the input vectors and the weights of the input and reservoir layer as shown in Equation (1).


(1) $$\begin{array}{l} \text{x}\left[n+1\right]=f^{\text{res}}\left({\text{W}}_{in}\text{u}\left[n+1\right]+{\text{W}}_{x}\text{x}\left[n\right]\right) \end{array}$$

where **u**[*n*] is the ESN input, **W**_in_ is the weight matrix between the input layer and reservoir layer, **W**_x_ is the weight matrix between the neurons within the reservoir layer, and *f*^res^ is the reservoir layer's activation function.

The states of the reservoir layer for all input vectors are used as an input to a supervised training to calculate the output weights **W**_out_. There are several linear regression methods to calculate the weights at the output layer. This work uses normal equation to implement the supervised training of the ESN (Figure 1B).


(2) $$W_{\text{out}}=(YX^{\prime }){\left(XX^{\prime }\right)}^{-1}$$

where **X** is a matrix concatenating all states of the reservoir layer and **Y** is a matrix of all training outputs.

The process for training the ESN can be explained through the following steps:

1. At initialization, randomly generate the weights for the input and reservoir layers (**W**_in_ and **W**_x_).
2. Drive the next input vector **u**[*n* + 1] to the input layer.
3. Calculate the response of the reservoir layer using (1).
4. Save the response in a matrix (**X**).
5. Repeat steps 2–4 for all input vectors.
6. Calculate output weights based on normal Equation (2).

Once the weights of the output layer are calculated, the network is ready and the state of the reservoir layer is used to calculate the output of the network as shown in Equation (3).


(3) $$\begin{array}{l} \text{y}\left[n+1\right]=f^{out}\left({\text{W}}_{out}\text{x}\left[n+1\right]\right) \end{array}$$

where **y**(*n* + 1) is the output of the network, **W**_out_ is the weight matrix at the readout layer and *f*^out^ is the readout layer's activation function.

## 3. Proposed ESN topology and architecture

ESN topology refers to the interconnection pattern between reservoir neurons^1^. Few topologies for reservoir were presented in the literature (Rodan and Tino, 2011). The ESN uses either fully or randomly connected reservoir layer topology (Jaeger, 2001). The shape of these topologies and their degree of connectivity is defined by the reservoir layer weight matrix **W**_x_, where the weights of the unconnected links are set to zero. Such a random method to generate topologies for the reservoir layer has design constraints. It requires several trials to find the appropriate topology for a given application. It is non-trivial to regenerate these connections dynamically; and it requires saving all the connections. There is no guarantee that the generated pattern will be the best choice for the target application (Rodan and Tino, 2011). Moreover, the highly connected random topology is too complex to implement in hardware where routing complexity, area overhead, and power consumption are significantly high. Thereby, simple reservoir topologies are desirable for hardware implementation of the ESN.

**Figure 2:**
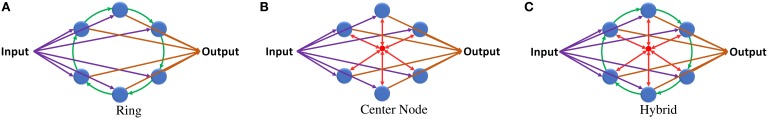
**(A)** ESN with ring reservoir topology. Each reservoir neuron has two inputs and one output. **(B)** ESN with center neuron topology. Reservoir neurons are connected to one center neuron that works as a hub. **(C)** ESN with hybrid topology. Each neuron has three inputs and one output.

In the ring topology presented in Rodan and Tino (2011), the reservoir layer neurons are connected in a ring shape where the output of each neuron is connected to only the neighboring neuron (Figure 2A). Equation (4) is used to calculate the state of a single neuron *x*(*s*) in the reservoir layer at a certain time step *n*. This equation can be generalized to calculate the state of the entire reservoir layer as shown in Equation (5).


(4) $$\begin{array}{l} \text{x}\left(s\right)\left[n\right]=f^{\text{res}}\left({\text{W}}_{in}\left(s\right)\text{U}\left[n\right]+{\text{W}}_{ring}\left(s\right)\text{x}\left(s-1\right)\left[n-1\right]\right) \end{array}$$

where **x**(*s*)[*n*] is the state of neuron *s* at time step *n*. **W**_*in*_(*s*) is the input weight associated with the neuron *s*. **U**[*n*] is the input at time step *n*. **W**_*ring*_(*s*) is the reservoir weight between the neurons *s* and *s* − 1. **x**(*s* − 1)[*n* − 1] is the state of the neuron *s* − 1 at the previous time step *n* − 1.


(5) $$\begin{array}{l} \text{X}\left[n\right]=f^{\text{res}}\left({\text{W}}_{in}\text{u}\left[n\right]+{\text{W}}_{ring}\overset{\gg }{\underset{\ll }{\text{X}\left[n-1\right]}}\right) \end{array}$$

where **X**[*n*] is the state of all neurons in the reservoir layer at time step *n*. **W**_*in*_ is the input weights matrix. **W**_*ring*_ is the reservoir weight vector (one weight for each neuron). $\overset{\gg }{\underset{\ll }{\text{X}\left[n-1\right]}}$ refers to the state of all neurons in the reservoir layer at time step *n* − 1 rotated by one.

This topology provides low degree of connectivity in the network but has high network diameter and average distance between reservoir neurons. For a ring reservoir layer that has *N* number of neurons the diameter is **N** − **1** and the average distance is $\frac{2}{3}\text{N}$. This high diameter causes delay in the response of the reservoir to the changes in the input. Such delay is undesirable in biosignal information processing applications.

Simple updates to the ring topology can fix the high diameter and average distance values. Adding different shortcut links in the reservoir layer decreases these values but it will result in an unbalanced network, where the distance between the neurons will vary depending on their location from the shortcut links. Using uniform connection links achieves constant distance between all neurons. Combining the ring topology, shown in Figure 2A, with the center neuron topology, shown in Figure 2B, may give the network balanced uniform shortcut links that increase the connections between neurons and improve network properties. Figure 2C shows the proposed hybrid reservoir topology. This topology has low diameter and average distance compared to the ring topology. The diameter is **2** and the average distance is less than **2**. The reservoir is tightly connected and is more sensitive to the changes in the input of the network. The state of the reservoir is calculated using Equations (6) and (7). Equation (6) is used to calculate the state of the center neuron which is used to calculate the state of the whole reservoir using Equation (7).


(6) $$\begin{array}{l} \text{X}\text{c}={\text{W}}_{up}\text{X}\left[n-1\right] \end{array}$$

where **Xc** is the state of the center neuron. **W**_*up*_ is the weight vector of synapses from the reservoir layer neurons to the center neuron.


(7) $$\begin{array}{l} \text{X}\left[n\right]=f^{\text{res}}\left({\text{W}}_{in}\text{u}\left[n\right]+{\text{W}}_{down}\text{X}\text{c}+{\text{W}}_{ring}\overset{\gg }{\underset{\ll }{\text{X}\left[n-1\right]}}\right) \end{array}$$

where **W**_*down*_ is the weight vector of synapses from the center neuron to the reservoir layer neurons.

In terms of complexity, the hybrid topology has two extra synapses per neuron compared to the ring topology. These synapses connect the neurons of the reservoir layer with the center neuron. The idea behind using the center neuron is to provide each neuron in the reservoir layer with information about the state of the whole reservoir. It emulates the fully connected topology in which each neuron has full access to all neurons within the reservoir layer.

**Figure 3:**
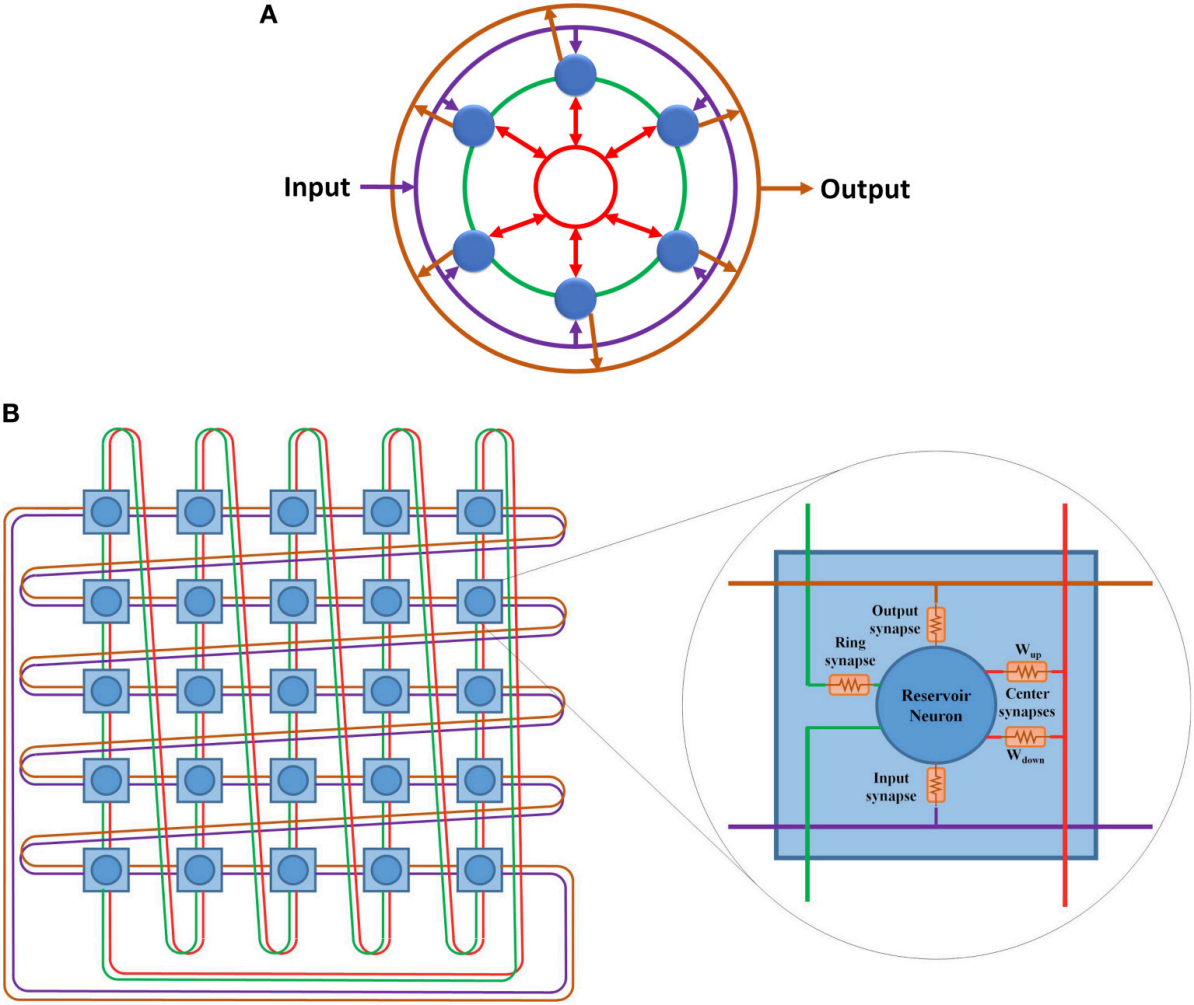
**(A)** Hybrid topology implemented as four rings. The rings are color coded based on Figure 2. Purple ring is used for the connections between the input layer and the reservoir layer. Green ring is used for the ring topology connections. Red ring is used for the center neuron connections. Orange ring is used for the connections between the reservoir layer and the output layer. **(B)** 5 *x* 5 doubly twisted toroidal network used to implement a 25 neurons hybrid reservoir. Dual links are used in this toroidal architecture to implement the four ring connection patterns required for the hybrid reservoir topology. The internal structure of each node in utilized toroidal architecture is shown. Each node contains a reservoir neuron along with all associated synaptic links.

The hybrid topology consists of four main groups of synaptic links: input set, output set, ring set, and center neuron set (see Figures 2C, 3). The input, output, and center sets connect each individual neuron in the reservoir to the three key neurons of the network: the input, output, and central neurons. Simultaneously, the ring set connects the reservoir neurons in a daisy chain (each neuron connects to its two neighbors in the ring). One link that passes through all the neurons in the reservoir layer in a ring shape can be used for each set to distribute these signals. This implies that four rings can implement all the required connections of the hybrid topology as shown in Figure 3A. This figure shows two types of ring connections. The first type is used for the input, output, and center neuron connections. These rings pass by the reservoir neurons which are connected to the rings through synaptic links. This means that each ring caries single electrical signal. The second ring type is used for the ring topology (the green ring). This ring pass through reservoir neurons. Any point of this ring has its own electrical signal that is different from any other point. The rings in Figure 3A are color coded to be consistent with Figure 2C that shows the hybrid topology.

### 3.1. Doubly twisted toroidal architecture

The doubly twisted toroidal architecture provides the ring connection pattern required for the hybrid topology shown in Figure 3B. This figure also shows the internal structure of each node in the proposed architecture. Each node in this architecture represents a neuron in the hybrid reservoir topology along with its four associated synapses: input, output, ring, and center neuron. The links in Figure 3B are color coded according to Figure 3A. Purple ring is used for the connections between the input neuron and the reservoir neurons. Orange ring is used for the connections between the reservoir neurons and the output neuron. Green ring is used for the ring topology connections. Red ring is used for the center neuron connections. Two types of synaptic links are used to connect reservoir neurons to the center neuron ring connection: **W**_*up*_ and **W**_*down*_. **W**_*up*_ synaptic links are used to carry signals from reservoir neurons to the center neuron. These signals are summed at the center neuron and then distributed back to the reservoir neurons using **W**_*down*_ synaptic links. For further information about the center neuron, refer to Equations (6) and (7).

In general, the doubly twisted toroidal architecture is reconfigurable and allows easy portability with few additional switching elements. An advantage in this case is the doubly twisted toroidal architecture's inherent connection pattern, which enables direct mapping of the hybrid topology without any additional switching elements. This makes the doubly twisted toroidal architecture simple compared to the other toroidal architectures, e.g., 2-D mesh, both in terms of the required resources and design complexity, where no multiplexers or switches are used to route the signals. One caveat is that the doubly twisted toroidal architecture configured for hybrid topology cannot be reused to implement other reservoir topologies. However, scalability is still provided by the twisted toroidal architecture where any size of reservoir can be implemented. There is a one-to-one relationship between the nodes in the doubly twisted toroidal architecture to the neurons in the reservoir. The total number of input links to each reservoir neuron is four (see Figure 3A). A node with a dual link doubly twisted toroidal architecture has eight links. Of the eight links, four links are used as input links to the neuron while the remaining are used as output links. The following two sections present the digital and mixed-signal implementations of this architecture.

## 4. Digital ESN design

The digital architecture of the ESN is optimized for reconfigurable platforms, without sacrificing its performance. The network operation was described fully parallel instead of partially sequential. This addresses the real-time input data streams, which have little delay associated with the consequent input and capture any spatial characteristics in the input data. Activation functions are designed with piece-wise approximations instead of using look-up tables. This design choice saves the total number of memory elements significantly, as the echo state network uses four weights per each reservoir neuron. In the high level model, the network functioned off of a delta cycle increment. The delta increment steps are simulation steps that do not advance nominal time and help in synchronizing all the parallel computations occurring within a timestep. For the digital design, registers were added between each neuron in the reservoir to produce constant results in simulation and hardware realization, otherwise, signals would constantly propagate throughout the reservoir asynchronously with untested behavior. The goal was to closely match the high level architecture model to the reconfigurable implementation so the analysis is valid for scaled networks.

**Figure 4:**
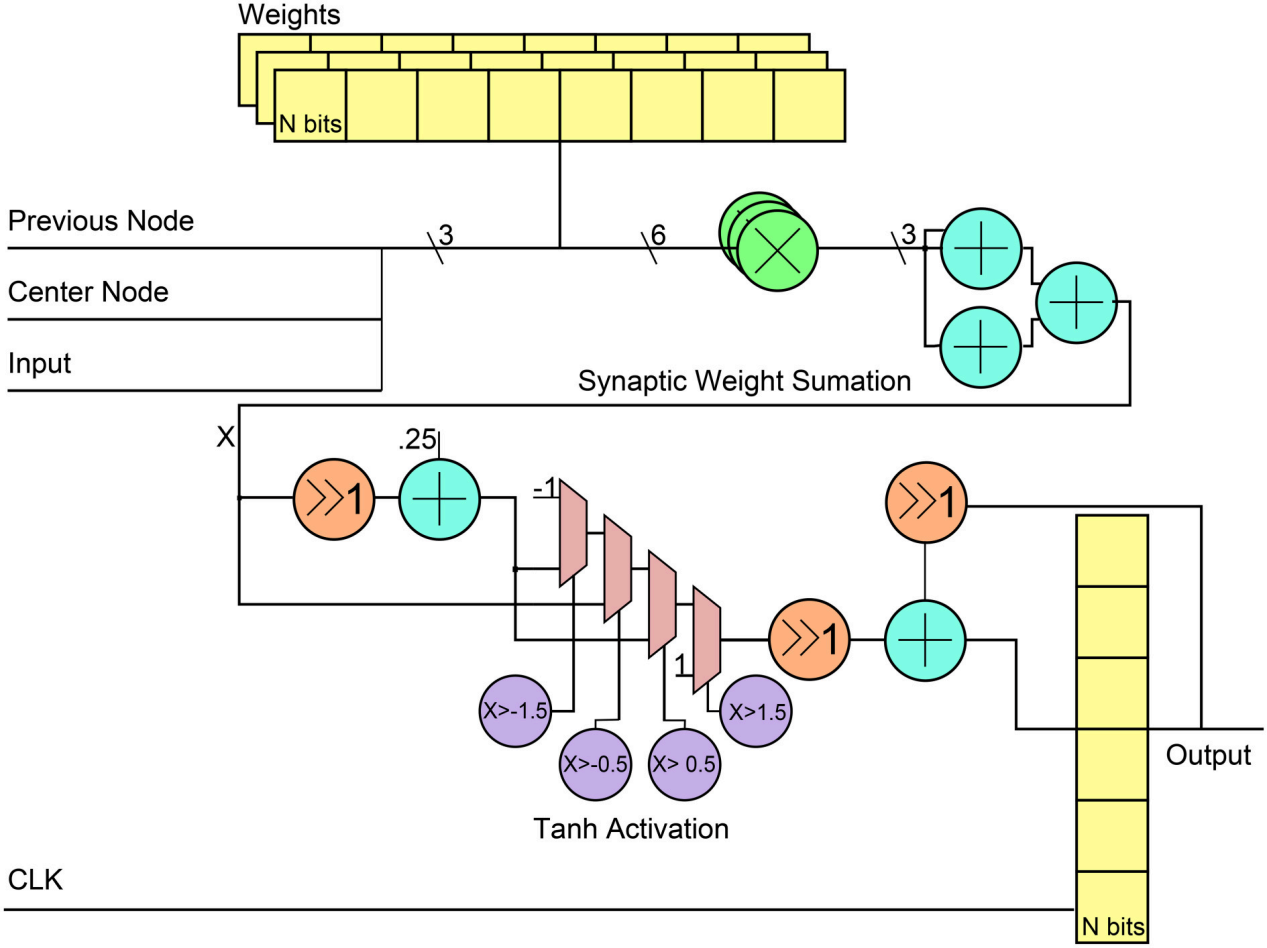
**RTL level diagram of a reservoir neuron in the digital ESN implementation**. All directions of signals are from left to right.

Figure 4 shows the general RTL diagram for a neuron in the network. Each neuron has three inputs that come from the network inputs, the previous neuron in the hybrid topology and a center neuron. The weights are stored in flip-flops and use a fixed point notation of 10 integer bits and 20 fractional bits, Q(10.20). These weights are multiplied by each output using Q(10.20) bit multipliers and then summed using 3 Q(10.20)-bit adders. For the neuron activation, the tanh function is used by implementing a piece-wise linear approximation that can be seen in Equation (8). The linear approximation was chosen to only have slopes that were powers of two to allow for shifts instead of multiplier units. Since the piece-wise function has five decision boundaries, three 2-1 Q(10.20) input MUXs were used as can be seen in the RTL diagram. The final step was applied to add a stronger temporal aspect to the network by making a neuron output a function of its current activation threshold and the previous activation threshold. This is done by summing the current neuron at time *t* with the previous result at time *t* − 1 after applying a scaling factor α such that **X**(**t**) = α**X**(**t**) + (α − 1)**X**(**t** − 1). The previous input is scaled by 1 − α while the current is scaled by α. For simplicity in hardware, the α-value was chosen to be 0.5 which allowed to shift right instead of using a multiplier before the fixed-point adder. The final registers are used to store the value to allow for this temporal calculation. Table 1 shows the resource utilization and power consumption for one reservoir neuron across three FPGA implementations with power broken down by synaptic multiplication, tanh activation, synaptic sum, and total.


(8) $$\begin{array}{l} {}[H]tanh(x)\;=\;1\;when\;x\;⩾\;1.5\\ \;=\;x/2+0.25\;when\;0.5\;⩾\;x>1.5\\ \;=\;x\;when\;-0.5\;⩾\;x>0.5\\ \;=\;x/2-0.25\;when\;-1.5\;⩾\;x>-0.5\\ \;=\;-1\;otherwise \end{array}$$

**Table 1:** **FPGA resource utilization and power consumption for hidden neuron on three FPGA**.

**FPGA**	**Clock (nS)**	**LUTs**	**FF**	**Mult(mW)**	**Tanh(mW)**	**Sum(mW)**	**Total(mW)**
Virtex5-LX110T	2.4	199	16	6.7	0.14	6.31	28.19
Virtex6-LX550TL	2.28	247	11	7.68	2.61	4.58	27.15
Spartan-6-LX150T	5.30	248	12	3.34	1.47	2.17	15.63

The output neuron is built in much the same way, except it takes as input the output from every neuron in the reservoir, not including the center neuron. As such, the addition tree used is log_2_(reservoirsize). Also, the activation function used is the linear output of the synaptic summation, so no additional logic is used for activation or the temporal aspect mentioned earlier.

## 5. Mixed signal design of the reservoir

The digital ESN design presented in the last section has good performance and is easy to port onto commercial off the shelf components (FPGAs). However, these realizations have large energy and area overheads owing to the limitations of the resource availability on the FPGAs. In this section, we propose an efficient ESN design that capitalizes on the inherent dynamics of simple mixed-signal circuits. First, we give a brief overview of the semi-empirical memristor device model that was used in this work. Then, we show that current-mode differential amplifiers operating in subthreshold have a natural hyperbolic tangent behavior. This result is well-known (Mead, 1989) and is summarized here for convenience. Then, we explore the possibility of using mismatch in closely-spaced transistors to implement the random weight distributions within the reservoir. This method is shown to be area efficient when the required weight resolution is high. Finally, we present a novel ESN readout layer based on a memristor crossbar circuit. The crossbar's high density enables full connectivity from the reservoir to the output layer at a low area cost.

### 5.1. Memristor device model

Memristors are two-terminal devices that have a state-dependent conductance (Chua, 1971, 2011; Chua and Kang, 1976). In this work, we use a semi-empirical memristor model derived from the works of Simmons (Simmons, 1963; Simmons and Verderber, 1967) and Mott and Gurney (1940). The memristor current is assumed to be dominated by tunneling, leading to the exponential I–V relationship:


(9) $$i_{m}=\{\begin{array}{c} \left(1-\gamma +g\gamma \right)G_{moff}\xi_{1}^{+}\text{sinh}\left(\frac{v_{m}}{\xi_{1}^{+}}\right),v_{m}\geq 0\\ \left(1-\gamma +g\gamma \right)G_{moff}\xi_{1}^{-}\text{sinh}\left(\frac{v_{m}}{\xi_{1}^{-}}\right),v_{m}<0 \end{array},$$

(10) $$\begin{array}{l} \frac{\Delta \gamma }{\Delta t}=\chi \left(v_{m}(t)\right)\\ \;=\{\begin{array}{cc} \xi_{4}^{+}\text{sinh}\left(\xi_{5}^{+}v_{m}(t)-\xi_{6}^{+}V_{tp}\right)f_{win}\left(\gamma \right), & v_{m}>V_{tp}\\ \xi_{4}^{-}\text{sinh}\left(\xi_{5}^{-}v_{m}(t)-\xi_{6}^{-}V_{tp}\right)f_{win}\left(\gamma \right), & v_{m}<V_{tn}\\ 0, & \text{otherwise} \end{array}, \end{array}$$

where $\xi_{i}^{+\left(-\right)}$ are fitting parameters, *V*_*tp*_ and *V*_*tn*_ are the positive and negative threshold voltages, and *f*_*win*_ is a window function that ensures γ does not become larger than 1 or smaller than 0. The window function is expressed as


(11) $$f_{win}\left(\gamma \right)=\{\begin{array}{cc} \text{exp}\left[-\xi_{2}^{+}\left(\gamma -\xi_{3}^{+}\right]\right)\frac{1-\gamma }{1-\xi_{3}^{+}}, & v_{m}\geq 0,\gamma \geq \xi_{3}^{+}\\ \text{exp}\left[\xi_{2}^{-}\left(\gamma -\xi_{3}^{-}\right)\right]\frac{\gamma }{\xi_{3}^{-}}, & v_{m}<0,\gamma \leq \xi_{3}^{-}\\ 1, & \text{otherwise} \end{array},$$

where $\xi_{i}^{+\left(-\right)}$ are fitting parameters. Empirical models similar to the one presented above are able to fit a wide range of device characteristics (Yakopcic et al., 2013). In this work, the model is fit to experimental results from W/Ag-chalcogenide/W devices which show reproducible incremental conductance switching (Oblea, 2010).

### 5.2. Reservoir neuron circuits

**Figure 5:**
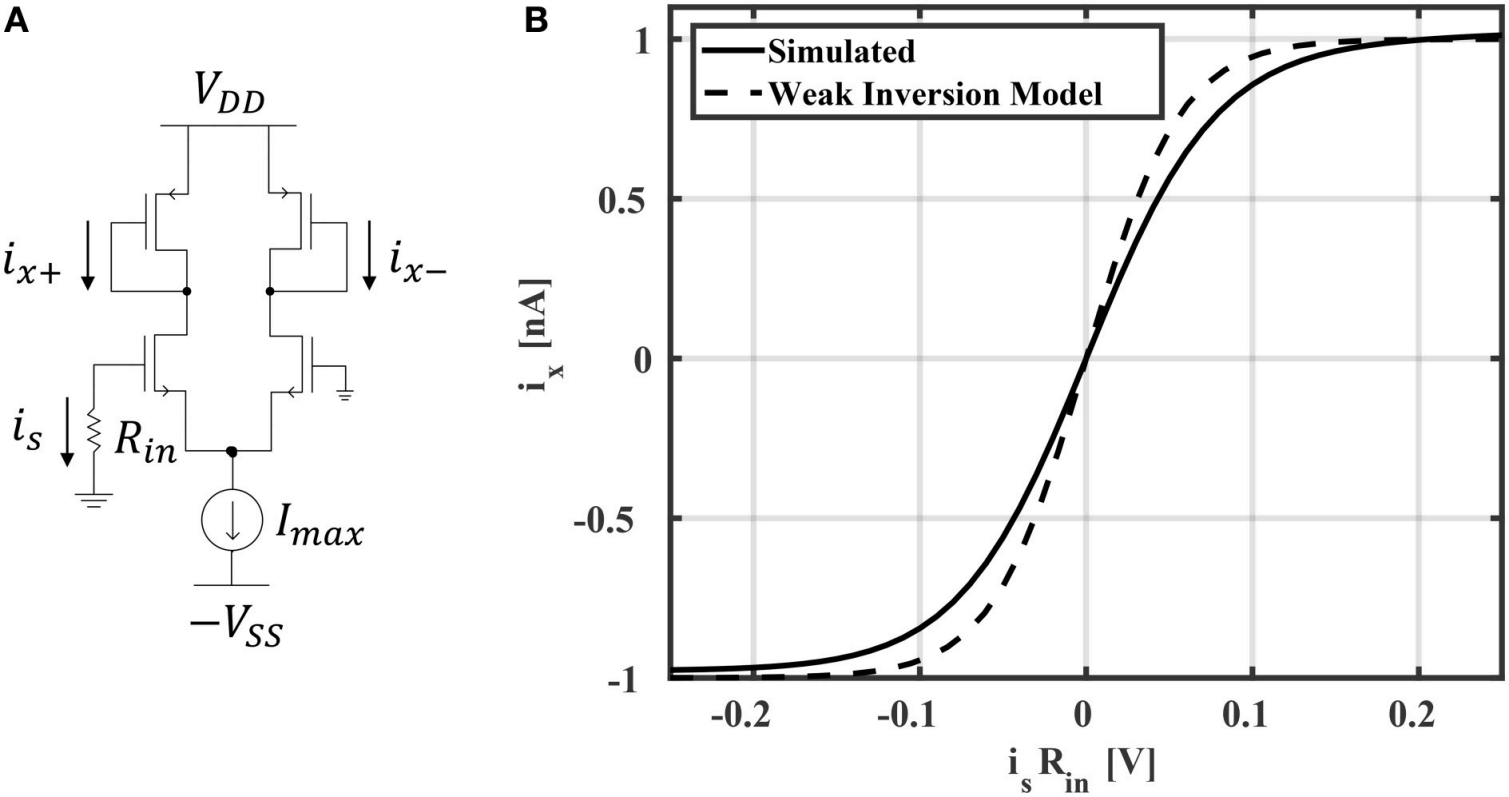
**(A)** Hyperbolic tangent neuron circuit and **(B)** its current-voltage relationship.

In general, reservoir computing methods may employ one of many different activation functions in the reservoir layer. ESNs generally use activation functions with sigmoid shapes, which may be unipolar (with a range between 0 and +1) or bipolar (with a range between –1 and +1). In multi-layer perceptron networks, it has been shown that bipolar functions, such as tanh, yield better accuracy (compared to unipolar activation functions) for classification tasks (Karlik and Olgac, 2010). In this work, we've found that tanh activation functions also result in richer reservoir dynamics leading to better classification accuracies in ESNs. Implementing a tanh activation function is straight forward using a differential amplifier biased in weak inversion (subthreshold; Mead, 1990). The neuron circuit schematic is shown in Figure 5A. It is easy to show that the neuron output is


(12) $$\begin{array}{l} i_{x}\equiv i_{x+}-i_{x-}=I_{max}tanh\left(\frac{i_{s}R_{in}}{2nV_{T}}\right), \end{array}$$

where *V*_*T*_ is the thermal voltage and *n* is a process-related constant which is ≈1.2 for 45 nm CMOS technology. In all of the simulations and results that follow, *I*_*max*_ is chosen to be 1 nA. The current-voltage relationship is shown in Figure 5B. The model in Equation (12) is compared to HSPICE simulation, showing good accuracy. Slight deviations from the model are likely due to short channel effects not considered in Equation (12). For example, a single-MOSFET current source with minimum sizing was used to reduce area, which results in channel length modulation. This effect could be reduced by increasing the current source's channel length or using a cascode mirror. However, this would result in an increased area cost. In total, the area of the neuron is


(13) $$\begin{array}{l} A_{neuron}\approx 5A_{match}, \end{array}$$

where *A*_*match*_ is the area of a MOSFET with a small standard deviation in its threshold voltage. Larger devices will have smaller standard deviations (Pelgrom, 1989). It can be shown that the standard deviation of *V*_*th*_ ≈ 14% when minimum sizing is used (*W* = 45 nm and *L* = 45 nm). In this work, we use a value of *A*_*match*_ that gives a standard deviation below 5%, which occurs when the device area is 10 × minimal sizing. Therefore, *A*_*match*_ ≈ 20250 nm^2^. In addition to the area, the neuron power consumption can be modeled as


(14) $$\begin{array}{l} P_{neuron}\approx I_{max}V_{DD}. \end{array}$$

Here, we have assumed that the neuron's static power dominates the total power consumption.

### 5.3. Reservoir synapse circuits

**Figure 6:**
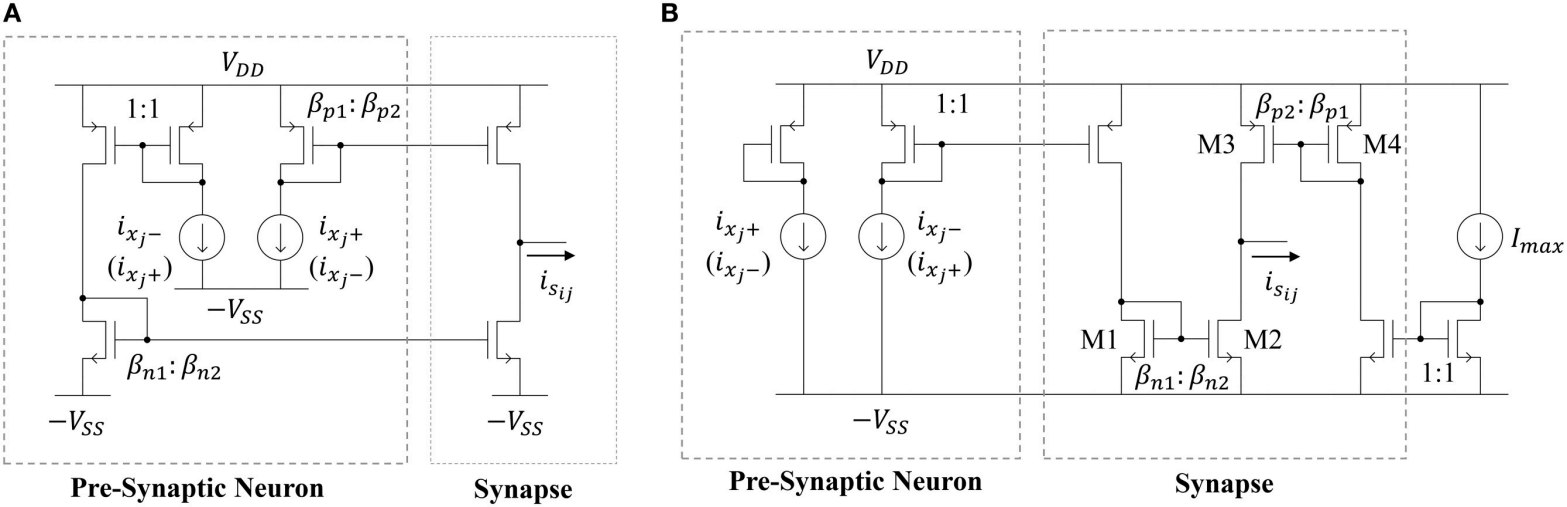
**Schematic of (A) a deterministic current-mode constant weight synapse circuit and (B) the proposed synapse circuit that leverages process variations**.

The most straightforward way to implement a synapse circuit compatible with the neuron described above is shown in Figure 6A. The synapse is a differential input, single-ended output design. The two inputs are driven by the scaled positive and negative outputs of the pre-synaptic neuron. Concretely, if the two PMOS mirror transistors have perfect matching properties, then the positive (negative) output of the pre-synaptic neuron *i*_*x*_*j*_+_ (*i*_*x*_*j*_−_) is scaled by a factor β_*p*2_ ∕ β_*p*1_, giving rise to the source current *i*_1_. Similarly, *i*_*x*_*j*_−_ (*i*_*x*_*j*_+_) is scaled by a factor β_*n*2_ ∕ β_*n*1_, giving rise to the sink current *i*_2_. Then, the output of the synapse *i*_*s*_*ij*__ is *i*_1_ − *i*_2_. If *w*_*ij*_ ≡ β_*p*2_ ∕ β_*p*1_ = β_*n*2_ ∕ β_*n*1_, then the synaptic output current will be


(15) $$\begin{array}{l} i_{s_{ij}}=w_{ij}i_{x_{j}}. \end{array}$$

Note that, if *i*_*x*_*j*_+_ (*i*_*x*_*j*__−) is mirrored through the PMOS mirror and *i*_*x*_*j*__ − (*i*_*x*_*j*__+) is mirrored through the NMOS mirror, then *w*_*ij*_ will be positive (negative). Therefore, one may randomly connect each synapse in the reservoir to the positive or negative output of a neuron (with both cases having 50% likelihood) to get a distribution of positive and negative weights. Furthermore, the size ratios β_*p*1_ ∕ β_*p*2_ = β_*n*1_ ∕ β_*n*2_ can be generated from a uniform or normal distribution to get the desired probability density function for the weights. The area cost of this approach depends on two factors. First, there will be a minimum sizing (*W*∕*L*)_*min*_ and related area *A*_*match*_ for each transistor that is governed by the desired level of matching in the current mirrors. The second factor governing the synaptic area cost is their weight distribution. Note that this distribution will be discrete since the weight values are determined by MOSFET geometry ratios. Let *w*_*res*_ be the resolution of the distribution. Furthermore, let each weight be a multiple of *w*_*res*_ such that *w*_*ij*_ = *k*_*ij*_*w*_*res*_, where *k*_*ij*_ ∈ ℕ (i.e., weights are evenly-spaced). Now, the expected synapse area for a particular weight distribution will be


(16) $$\begin{array}{l} E\left[A_{synapse}^{Baseline}\right]=2A_{match}E\left[\frac{k_{ij}}{\text{gcd}\left(\frac{1}{w_{res}},k_{ij}\right)}\right], \end{array}$$

where gcd(·) is the greatest common divisor. Equation (16) accounts for the fact that the minimum synapse size will be 2*A*_*match*_, since the circuit is composed of two MOSFETs. It also takes into account that the sign of the input MOSFETs of the two current mirrors that define the synapse will affect the synaptic area. For example, consider the case where the weight resolution is *w*_*res*_ = 1/100. For a weight to have a value of *w*_*ij*_ = 1/100, the input MOSFETs should have areas of 100*A*_*match*_, while the output MOSFETs have areas of *A*_*match*_. For the same resolution, to have a weight of *w*_*ij*_ = 1, the input and output synapses could both have areas of 100*A*_*match*_, but it is much more area-efficient for them to all have areas of *A*_*match*_. In other words, the area assumed in Equation (16) is the minimum possible area to get the desired weight values and meet the minimum area requirement for matching.

Unfortunately, Equation (16) indicates that it is not feasible, due to the large area impact, to implement high-resolution weights using the circuit in Figure 6A. Instead, our approach is to leverage the effects of transistor mismatch to achieve a random weight distribution in the ESN input and reservoir layers. This idea has been explored previously by Yao et al. (2013) in an extreme learning machine (ELM) implementation with unipolar input (inputs to the synapse are strictly positive or negative) synapses. Here, we expand on their work by (i) proposing a bipolar input synapse that leverages mismatch and (ii) analyzing the impact of random mismatch-based synapses on the area and power of the network. The proposed circuit is shown in Figure 6B. Similar to the synapse circuit in Figure 6A, the proposed design has a differential input and single-ended output. However, now the two inputs are driven by the scaled positive or negative output of the pre-synaptic neuron, as well as the scaled maximum current *I*_*max*_. The circuit leverages random mismatches in the transistor threshold voltages *V*_*th*_ and gain factors β due to process variations. All of the circuits operate in subthreshold, so MOSFET drain currents are exponential in the threshold voltage and linear in the gain factor. Therefore, we can simplify our analyses by only considering variations in threshold voltage. For the NMOS current mirror in Figure 6B, we define *w*_1_*ij*__ as


(17) $$\begin{array}{l} w_{1_{ij}}\equiv \frac{i_{out}}{i_{in}}=\frac{i_{s_{ij}-}}{i_{x_{j}-}}=\frac{\beta_{n2}}{\beta_{n1}}e^{\frac{\Delta V_{th}}{nV_{T}}} \end{array}$$

where Δ*V*_*th*_ is a Gaussian random variable that quantifies the difference between two closely-spaced transistors' threshold voltages, *n* is a process-related constant which is ≈1.2, and *V*_*T*_ is the thermal voltage (≈26 mV at room temperature). Note that, by definition, *w*_1_*ij*__ will be lognormally distributed. Similarly, for for the PMOS mirror, we define *w*_2_*ij*__ as


(18) $$\begin{array}{l} w_{2_{ij}}\equiv \frac{i_{out}}{i_{in}}=\frac{i_{s_{ij}+}}{I_{max}}=\frac{\beta_{p2}}{\beta_{p1}}e^{\frac{\Delta V_{th}}{nV_{T}}}, \end{array}$$

where *w*_2_*ij*__ is also lognormally distributed. Finally, combining Equations (17) and (18) yields


(19) $$\begin{array}{l} i_{s_{ij}}=i_{s_{ij}+}-i_{s_{ij}-}=w_{2_{ij}}I_{max}-w_{1_{ij}}i_{x_{j}-}. \end{array}$$

Now, we'd like to express *i*_*s*_*ij*__ in terms of a single weight value. Using the fact that *I*_*max*_ = *i*_*x*_*j*_+_ + *i*_*x*_*j*_−_, we can rewrite Equation (19) as


(20) $$\begin{array}{rcl} i_{s_{ij}} & = & \left(\frac{1}{2}w_{1_{ij}}+\Delta w\right)\left(i_{x_{j}+}+i_{x_{j}-}\right)-w_{1_{ij}}i_{x_{j}-} \end{array}$$

$$\begin{array}{rc} = & \frac{1}{2}\left(i_{x_{j}+}-i_{x_{j}-}\right)w_{1_{ij}}+I_{max}\Delta w=i_{out}w_{ij}+I_{max}b_{ij}. \end{array}$$

Now, the synaptic output current is written as the pre-synaptic neuron's output times a lognormally distributed weight value plus a bias term. The bias term *b*_*ij*_ = *w*_2_*ij*__ − 0.5*w*_1_*ij*__ will be distributed as the difference of two lognormally distributed random variables.

**Figure 7:**
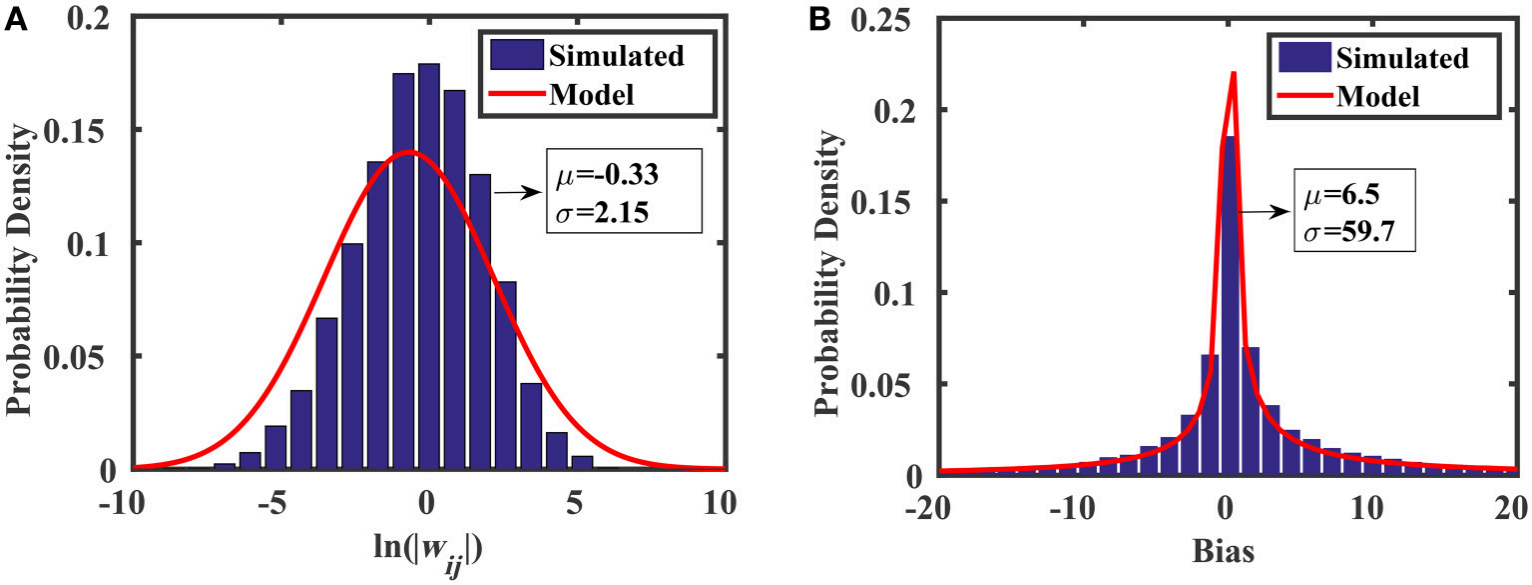
**Monte Carlo analyses showing the distributions of (A) random weights and (B) random biases associated with the synapse design in Figure 6B**.

The random weight distribution within the reservoir can be adjusted by modifying the sizes and gain factor ratios for transistors M1–M4. Summarizing the analysis above, and assuming we have


(21) $$\begin{array}{l} |w_{ij}|\sim lnN\left(ln\frac{\beta_{n2}}{2\beta_{n1}},\frac{A_{V_{th}}^{2}}{WL}\right) \end{array}$$

where lnN is the lognormal distribution, and *A*_*V*_*th*__ is a constant that determines the standard deviation of threshold voltage mismatch in closely-spaced transistors (Pelgrom, 1989). For the 45 nm low power predictive technology model (PTM) used in this work, *A*_*V*_*th*__ = 4 mV·μm. We see from Equation (21) that the spread of the weight distribution (as well as the bias distribution) can be tuned by increasing the area of the transistors used in the current mirrors. Figure 7 shows the distributions of weights and biases in the reservoir using when transistors are minimally sized (i.e., *W* = *L* = 45 nm). The simulated results were produced using 10,000 Monte Carlo simulations in HSPICE, where each of the two current mirrors in Figure 6B (M1–M4) had randomly mismatched threshold voltages with 0 mean and a variance of $A_{V_{th}}^{2}∕\left(WL\right)$. The distribution models (discussed above) show excellent agreement with the simulations.

**Figure 8:**
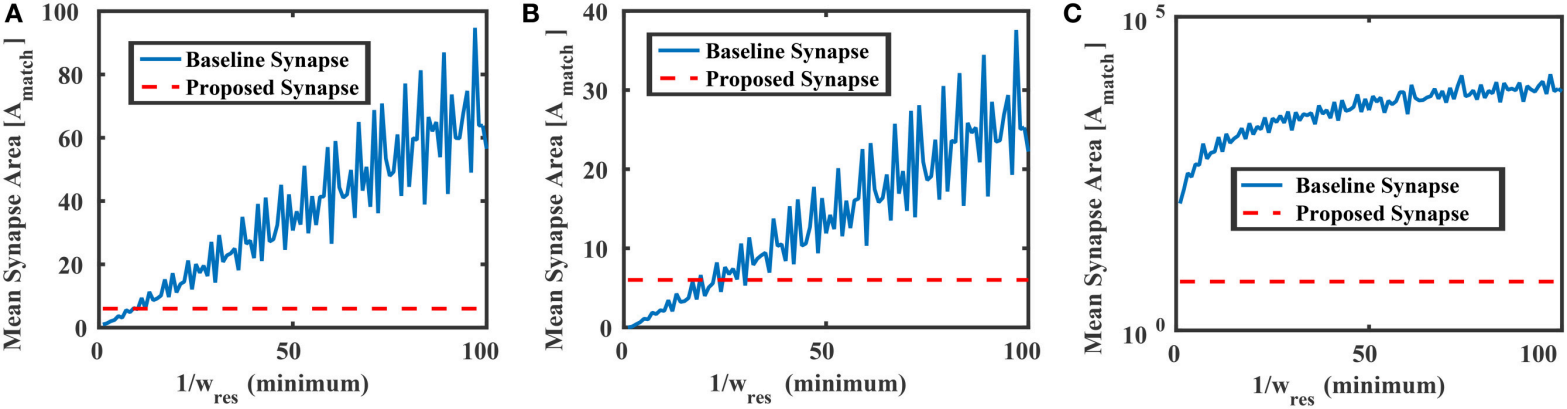
**Mean areas vs. the weight resolution for the baseline and proposed synapses circuits in Figure 6 where the baseline design was distributed (A) uniformly between −1 and +1, (B) normally with 0 mean and std = 0.1, and (C) lognormally with 0 mean and std = 2.85**. In each case, the proposed synapse design's maximum area (6*A*_*match*_) is shown as a reference.

The area of the proposed synapse is


(22) $$\begin{array}{l} A_{synapse}^{Proposed}=2A_{match}+4A_{var}=A_{match}\left(2+4a\right), \end{array}$$

where *A*_*var*_ is the area of the transistors M1–M4, and *a* ≡ *A*_*var*_ ∕ *A*_*match*_. Figure 8 compares the mean area of the baseline synapse and the proposed synapse for three different weight distributions over multiple resolutions. In each case, the maximum area of the proposed synapse occurs when *A*_*var*_ = *A*_*match*_ (*a* = 1), resulting in an area of 6*A*_*match*_. In Figure 8A, the weights of the baseline synapse are distributed uniformly between –1 and +1. Note that the roughness in the baseline curves comes from the gcd in Equation (16). For low weight resolutions (e.g., 1/*w*_*res*_ < 10), the baseline design is actually more area efficient than the proposed variation-based synapse. However, as the weight resolution increases, the baseline synapse's area becomes very large. Similar results are shown for the cases when the baseline weights are distributed normally (μ = 0, σ = 0.1) and lognormally (μ = 0, σ = 2.85), as shown in Figures 8B,C, respectively. In addition to the area, we can also model the power consumption of the proposed synapse circuit, which will be


(23) $$\begin{array}{l} \begin{array}{cc} P_{synapse_{ij}}= & \left(V_{DD}+V_{SS}\right)I_{max}\eta_{j} \end{array}\\ +\;\left(v_{s_{i}}+V_{SS}\right)I_{max}\eta_{j}w_{1_{ij}} \end{array}$$

$$\begin{array}{l} +\;\left(V_{DD}-v_{s_{i}}\right)I_{max}w_{2_{ij}} \end{array}$$

$$\begin{array}{l} +\;\left(V_{DD}+V_{SS}\right)I_{max} \end{array}$$

$$\begin{array}{l} +\;v_{s_{i}}\left(I_{max}w_{2_{ij}}-I_{max}\eta_{j}w_{1_{ij}}\right), \end{array}$$

where *v*_*s*_*i*__ is the voltage at the synapse output node (input to the post-synaptic neuron) and η_*j*_ is the activity factor of the pre-synaptic neuron, which can be estimated as 0.5. The first four terms account for the currents flowing through M1, M2, M3, and M4, respectively (Figure 6B). The last term accounts for the current flowing into the post-synaptic neuron. Recall that the neuron's input stage is a resistor with one terminal grounded. Combining like terms leads to a simplified expression for the power consumption given as


(24) $$P_{synapse_{ij}}=V_{DD}I_{max}\left(2\eta_{j}+\eta_{j}w_{1_{ij}}+w_{2_{ij}}+2\right).$$

Here, we have used the fact that *V*_*DD*_ = *V*_*SS*_.

### 5.4. Readout layer and training circuits

**Figure 9:**
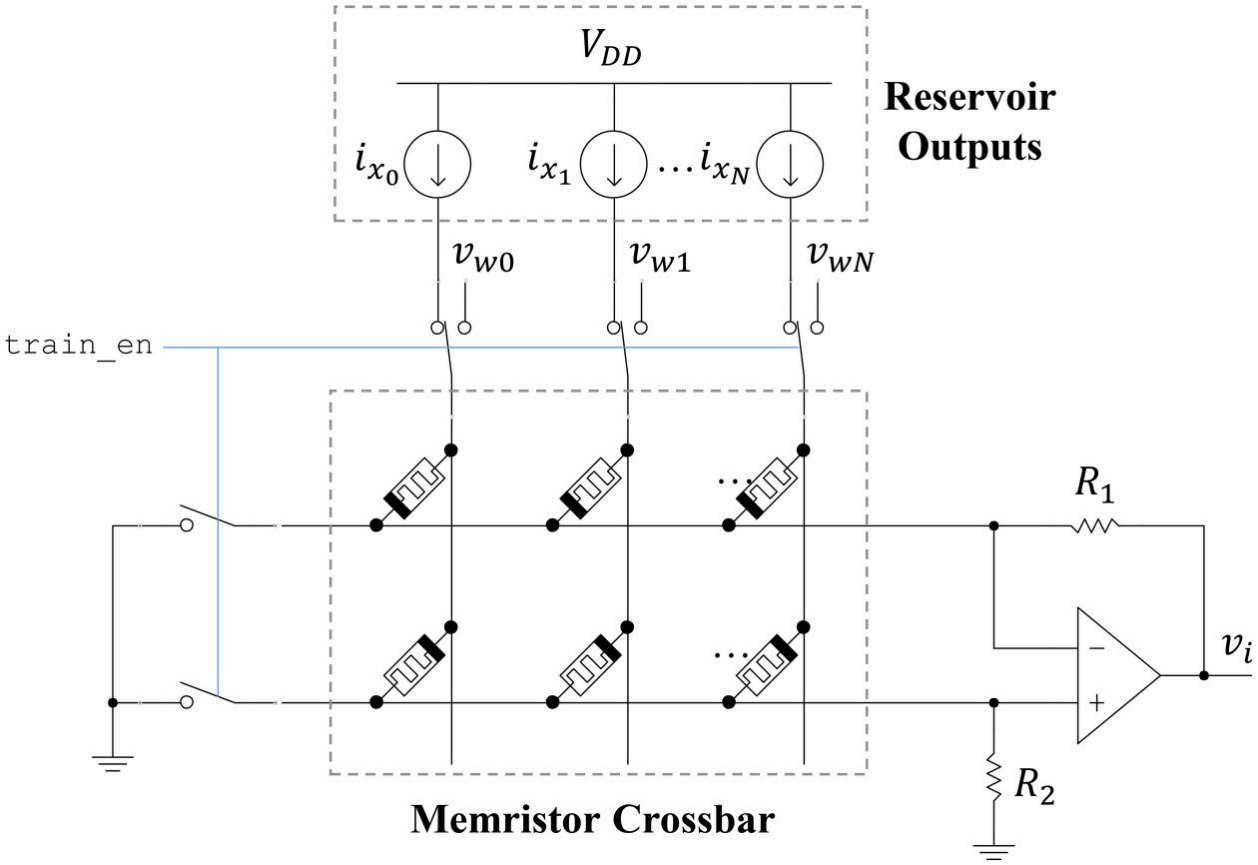
**Readout circuit for a single ESN output**. A memristor crossbar provides trainable weights to the linear output neuron.

The ESN readout layer leverages the non-volatility and plasticity of memristors to store and adjust the output weight values. The circuit connecting the reservoir to an output neuron is shown in Figure 9. A memristor crossbar is used to implement the synaptic weights at a low area cost. There are two memristors for each reservoir output, represented as ideal current sources. One memristor (top row) inhibits the output, while the other one (bottom row) excites the output. This allows each synaptic weight to achieve both positive and negative weight states. If we let *R*_1_ = *R*_2_ = *R*, then the output voltage *v*_*i*_ is given as


(25) $$v_{i}=\sum_{j\;=\;0}^{N}i_{x_{j}}w_{ij},$$

where *w*_*ij*_ is


(26) $$w_{ij}=\frac{G_{m+j}-G_{m-j}}{G_{m+j}+G_{m-j}}R.$$

Here, *G*_*m*+*j*_ refers to the bottom-row memristor for a particular input column *j*. Similarly, *G*_*m*−*j*_ refers to the top-row memristor for a particular input column *j*. The memristor states can be adjusted by connecting each crossbar row to ground and each crossbar column to a write voltage *v*_*wj*_. Pulses are applied to the columns using the stochastic least-mean-squares (SLMS; Merkel and Kudithipudi, 2014) algorithm to train the output layer. The area of the output layer can be approximated as


(27) $$A_{output}\approx NMA_{match}+9A_{match}.$$

In Equation (27), *M* is the number of ESN outputs. Here, we have assumed a seven-transistor opamp (differential stage followed by a common source gain stage). The resistances in the opamp circuit can be implemented using memritors to reduce the on-chip area cost. Furthermore, we assume that the reservoir outputs and opamp will dominate the total area. Finally, the power consumption for the output layer is


(28) $$P_{output}\approx \eta NMI_{max}V_{DD}+2MI_{bias}V_{DD}.$$

The first term is from the reservoir outputs feeding into the output layer weights. The second term is for the opamp, where *I*_*bias*_ = 0.1μA, which is used to bias both stages. The area and power of the circuits discussed in this section are compared to a digital (FPGA) implementation in Section 7.

## 6. Biosignal benchmarks

Electroencephalogram (EEG) and Electromyogram (EMG) are the two bioelectrical signals used in this study. Datum for both these signals is collected using sensors and differential amplifiers. The case studies we explored for these two signals are epileptic seizure detection (EEG) and control of prosthetic fingers using EMG. However, the proposed models are generic and can be applied to other disorders and therapeutic diagnostic studies. Epilepsy is the fourth most common neurological disorder, where one in 26 people will develop this disorder at sometime in their life (Sirven, 2015). There are a few therapeutic interventions possible for treating seizures. However, detecting the onset of a seizure, by automatic monitoring of EEG data, will aid the doctors/emergency responders to provide appropriate drug dosage based on the remaining epileptic activity. The common pattern in the case of seizures is that the brains electrical signals repeat themselves (Sirven, 2015). The EEG dataset we have used in this research was presented in (Andrzejak et al., 2001). It consists of 500 single-channel EEG segments of 23.6 s recorded at sampling rate of 173.61 Hz. The dataset was divided into five sets (denoted A–E), each set contains 100 EEG segments. The set A, which contains surface EEG recordings of five healthy volunteers, and E which contains seizure activity segments taken from five patients, were used in this research. The dataset is publicly available at (Andrzejak et al., 2001).

Electromyography (EMG) is a medical procedure to measure and record the action potential of the skeletal muscles which are neurologically or electrically stimulated (Keenan et al., 2006). The EMG contains information about the physical state of the neuromuscular system such as unit recruitment and firing and motion intention (Keenan et al., 2006). This work classifies EMG signals based on arm finger motion. The idea is to use the resulting information as input signals for prosthetic finger control. The EMG dataset used in this project is presented in Khushaba and Kodagoda (2012). It contains surface EMG signals recorded from six male and two female subjects aged between 20 and 35 years. These are healthy volunteers with no neurological or muscular disorders. The EMG signals were recorded while the subjects were moving their limbs and fingers according to a predefined procedure. Eight surface EMG electrodes were used to collect the data. The electrodes were placed across the circumference of the forearm. The signals were amplified to a total gain of 1000. They are sampled using 12-bits ADC at sampling rate of 4 *kHz*. The signals are band filtered between 20 and 450 Hz. The dataset is divided into 15 classes based on finger movements. It contains three EMG segments of each subject per class. Five classes representing individual finger movements are used in this project.

## 7. Results and analysis

### 7.1. Epileptic seizure detection

Two hundred EEG segments (160 for training and 40 for testing) are used for epileptic seizure detection. The normalized absolute values of these segments are fed into the ESN. The output of the ESN is compared against a threshold value to calculate the final binary output.

**Figure 10:**
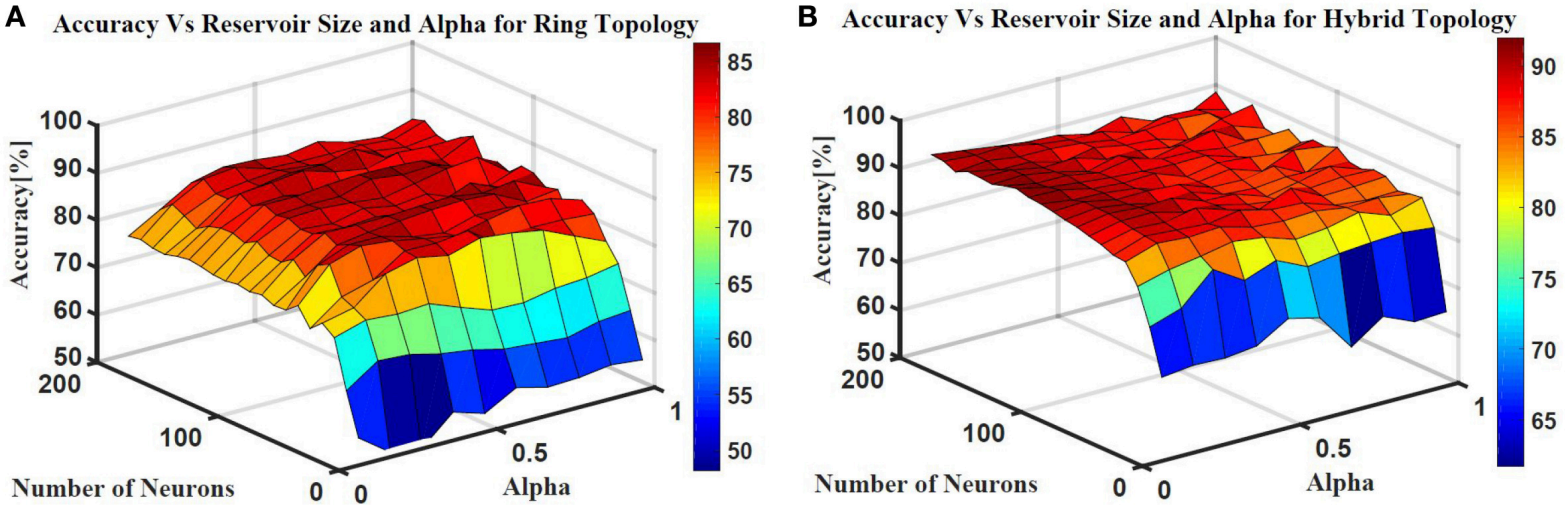
**Epileptic seizure detection accuracy vs.the reservoir size and alpha for (A) Ring topology with maximum accuracy of 86% and (B) Hybrid topology with maximum accuracy of 90%**.

Two ESN topologies are used for epileptic seizure detection: ring topology and hybrid topology. Several simulations were conducted for each topology to find the best reservoir size and alpha value that give the highest classification accuracy. The accuracy is calculated as a ratio of the time the output is correct to the total simulation time. Figure 10 shows the testing accuracy vs. reservoir size and alpha for the ring and random topologies. In both topologies, the accuracy increases as the reservoir size increases. However, the accuracy stabilizes within a range once the reservoir size exceeds 100 neurons. Results also showed that changing alpha value does not have as much affect on the accuracy. In general, alpha value of 0.5 works for the two topologies. The maximum accuracy achieved is ≈86 and ≈90% for the ring and hybrid topology, respectively. The extra synaptic connections within the reservoir layer of the hybrid topology helps increasing the accuracy. These connections increase signal exchange between reservoir neurons and provide more information about the overall situation of the reservoir to each neuron which increases the response of the reservoir to the changes in the input.

Three FPGA devices were utilized for testing the RTL design of the ESN architecture. The RTL implementation of the ESN has 30 neuron with a fixed point format of Q(10.22). The hardware model achieved 67.4% accuracy averaged over 10 runs at 30 neurons in the reservoir. This accuracy is comparable to the accuracy achieved in the behavioral model that has the same size. Table 2 shows the clock and resource utilization of three different tested FPGA devices.

**Table 2:** **FPGA resource utilization for 30 neuron reservoir implementation on three different FPGAs**.

**FPGA**	**Clock (ns)**	**LUTs**	**Flip-Flop**	**DSP**	**Utilization**	**Power (mW)**
Virtex5-LX110T	44.782	50,698	2894	150	73%	125.36
Virtex6-LX550TL	37.960	12,946	2900	150	5%	47.76
Spartan-6LP-LX150T	84.221	13,173	2907	150	14%	26.7

### 7.2. Prosthetic finger control

**Figure 11:**
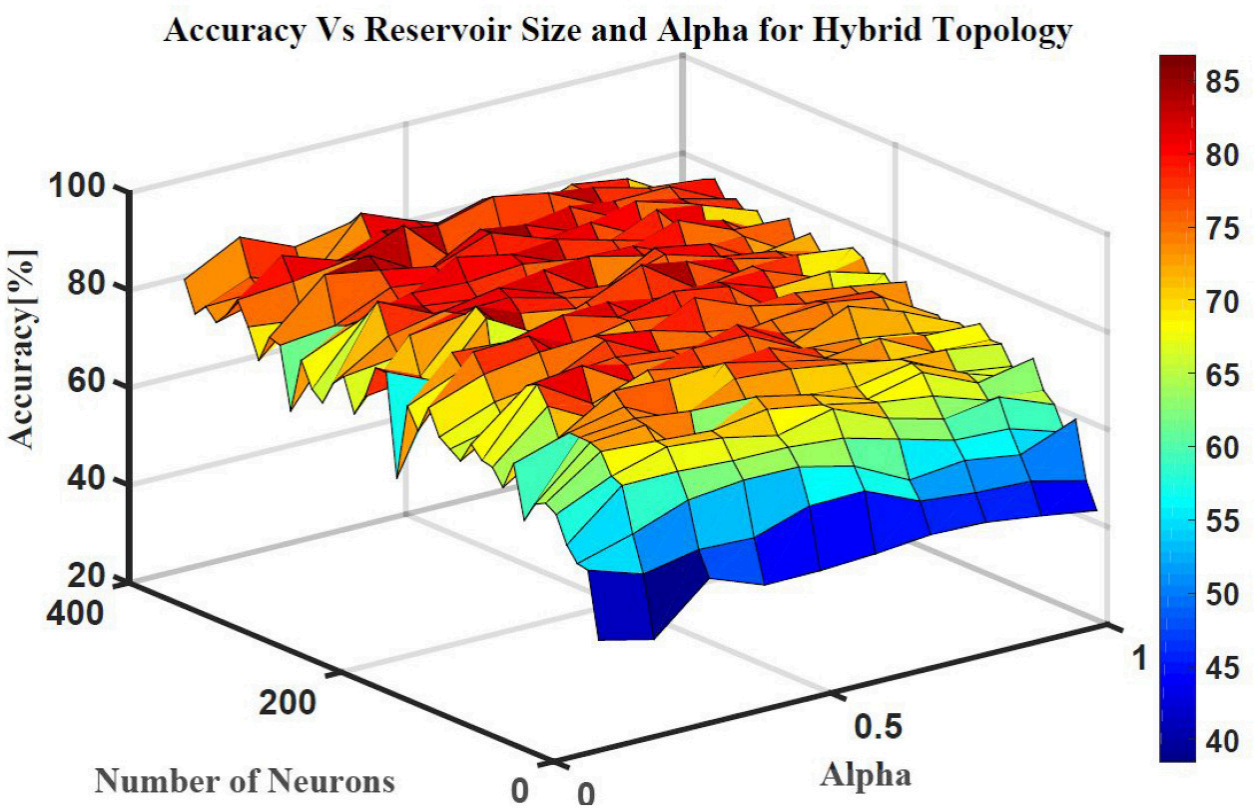
**The effects of the number of neurons within the reservoir and alpha on the testing accuracy of finger motion recognition using hybrid topology**.

**Figure 12:**
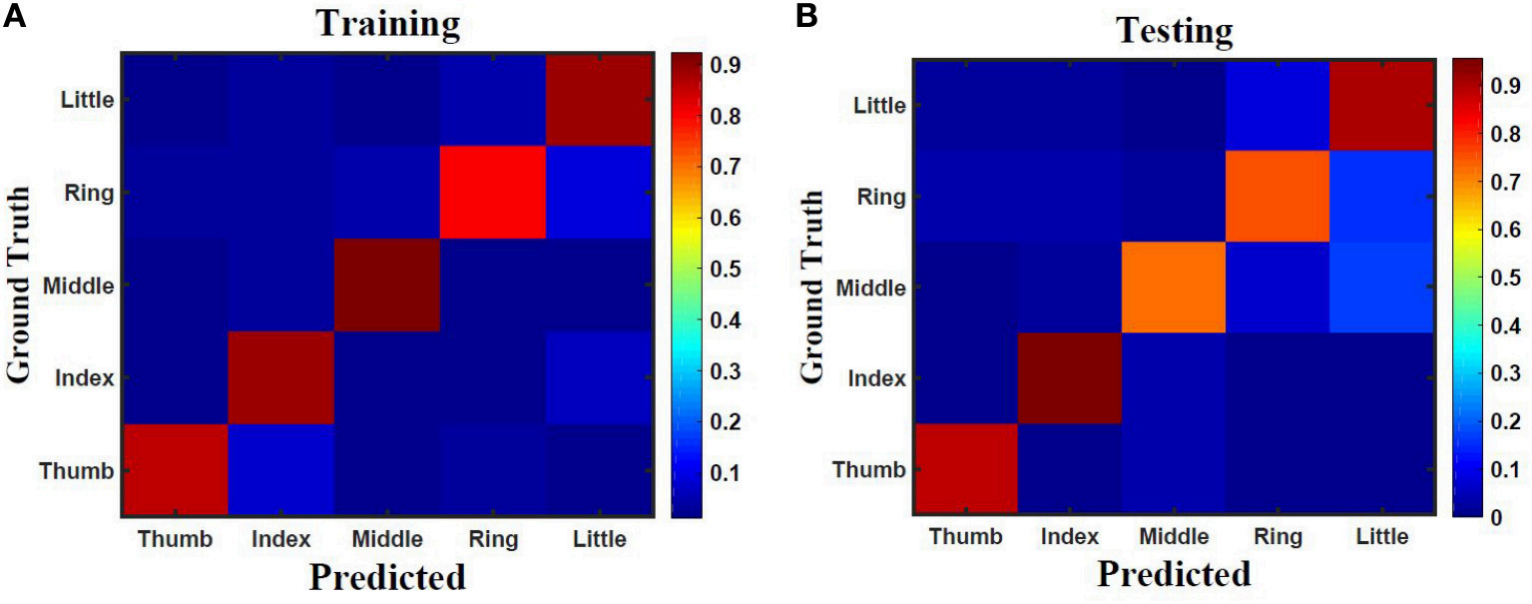
**Confusion matrix of finger classification from surface EMG signals using 300 neurons hybrid reservoir for (A) training with accuracy of 87% and (B) testing with accuracy of 84%**.

Five classes of individual finger motions are used in this work. Each class contains 24 EMG segments of length 20 s. The segments are divided into smaller parts of length 4 s. This will increase the total number of segments to 120 per class. Hundred segments are used for training while the rest 20 segments are used for testing. The hybrid ESN topology is used for finger motion classification. Eight input neurons are used (one neuron for each EMG channel) while five neurons are used for the output (one neuron per class). The output signals of these neurons are processed using winner-take all method to calculate the final binary output. The performance of the hybrid topology was analyzed to find the best parametric values. Several simulations were conducted over varied reservoir size and alpha values. The accuracy is calculated as a ratio between the number of trails the classification is correct to the total number of trails. Figure 11 shows the test accuracy vs. reservoir size and alpha. The average accuracy of 10 trials for each combination of size and alpha is showed in this Figure. The accuracy increase as the size of the reservoir increase for the sizes lower than 300 neuron. However, the accuracy stabilizes in a range for the larger reservoir sizes. The maximum training and testing accuracy achieved is 87 and 84%, respectively. Figure 12 shows confusion matrices of classification accuracy of training and testing.

### 7.3. Metrics for evaluating ESN

The performance of the reservoir is dependent on the randomly generated weights of the reservoir and several other parameters such as alpha and reservoir size. Finding the best values for these parameters has been an open question. The overall performance of the reservoir has been used to study effect of these parameters. This requires a complete training of the reservoir for a specific application, which takes long time and requires more processing resources. Further more the best parameter values may vary depending on the targeted application. More general metrics that are independent from the target output are required to test the reservoir. These reservoir metrics are measurements of the quality of the reservoir. Several metrics have been proposed in the literature (Gibbons, 2010; Norton and Ventura, 2010). Chrol-Cannon et al. (Chrol-Cannon and Jin, 2014) compared the ability of four reservoir metrics to measure the performance of several reservoir topologies. The reservoir metrics used in their study are: class separation, kernel quality, Lyapunov's exponent, and spectral radius. Results show that kernel quality and Lyapunov's exponent strongly correlate with reservoir performance. These two metrics are used in this work for reservoir sizes varying from 10 to 100 neurons, over 100 trials.

### 7.4. Kernel quality

Kernel quality is a measure of the linear separability of the reservoir. It is first presented in Legenstein and Maass (2007) and revisited by Busing Büsing et al. (2010), and Chrol-Cannon et al. (Chrol-Cannon and Jin, 2014) as a practical reservoir metric. The reservoir response to the whole set of input vectors is used to calculate this metric. The whole reservoir states are concatenated in a matrix M where each column in M represents reservoir response to one input vector. The kernel quality is calculated by taking the rank of this matrix. It represents the network freedom to represent each input stimulus differently. The target kernel value is equal to the size of the reservoir which means that each reservoir neuron generates its unique response that can't be regenerated by using linear combinations of the responses of the other neurons. Reservoirs with optimal separability will have a high kernel quality.

**Figure 13:**
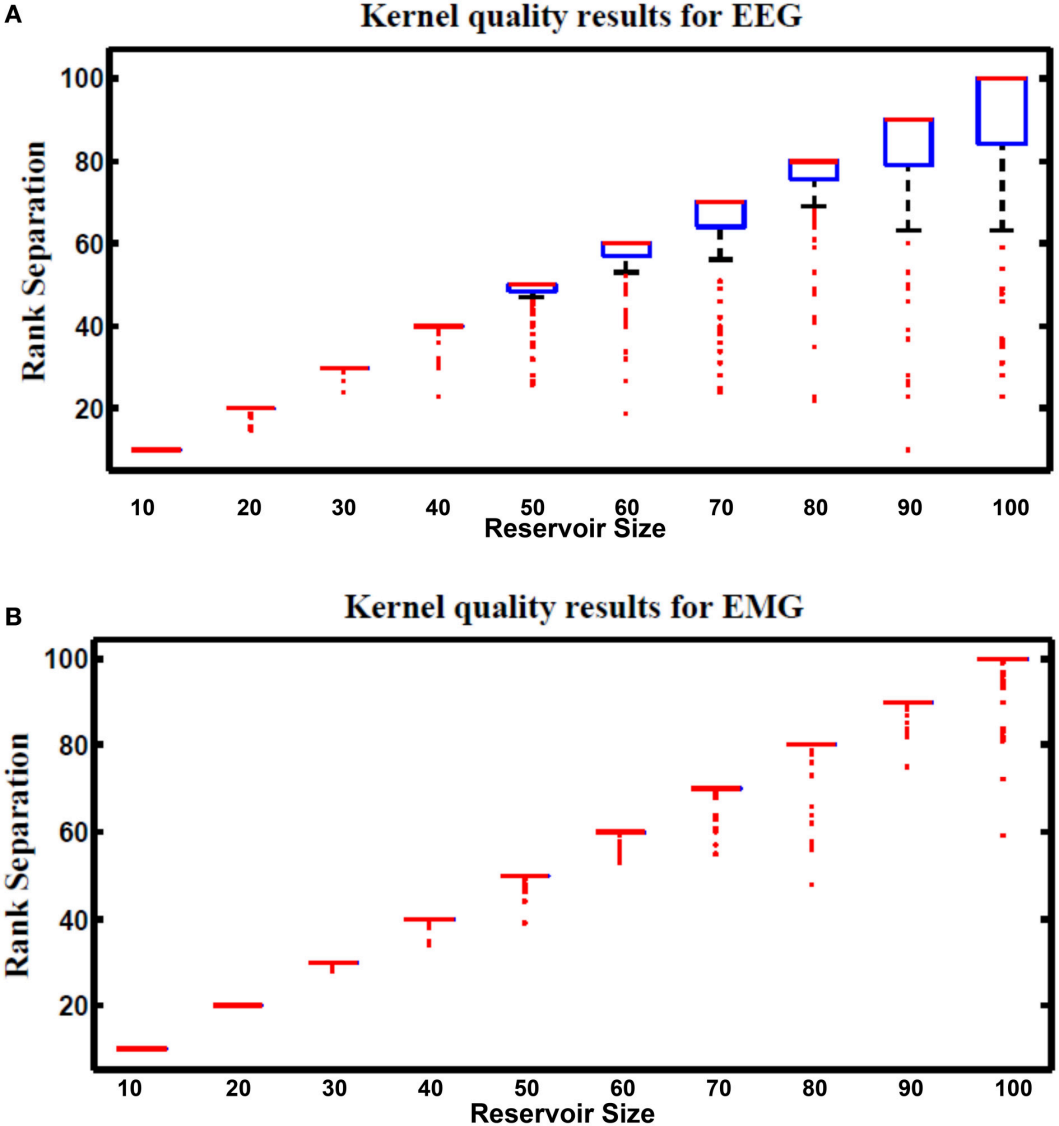
**Kernel results vs. different reservoir sizes for hybrid topology reservoir testing (A) EEG and (B) EMG signals**. The boxes represent the 25th and 75th percentiles of the measurements. The whiskers represents values out of this 25–75th percentiles. Since the percentiles range is centered in **(B)**, the sizes of the boxes in **(B)** are very small (just a line that represent the mean of the range).

Figure 13 shows kernel quality results for the hybrid reservoir topology processing EEG and EMG signals. The median of kernel quality values are close to the target for different reservoir sizes for both EEG and EMG signals. Results show that there is variation in the kernel quality values especially for reservoir sizes larger than 50 neurons. This variation is a result of the randomly generated weight for the input and reservoir synapse where low kernel quality value could be a result of unsuitable set of random weights. In general, the kernel quality value for EMG data seems more stable compared to that of the EEG data. The nature of the input signals can be leading to this slight difference between the EEG and EMG.

### 7.5. Lyapunov's exponent

**Figure 14:**
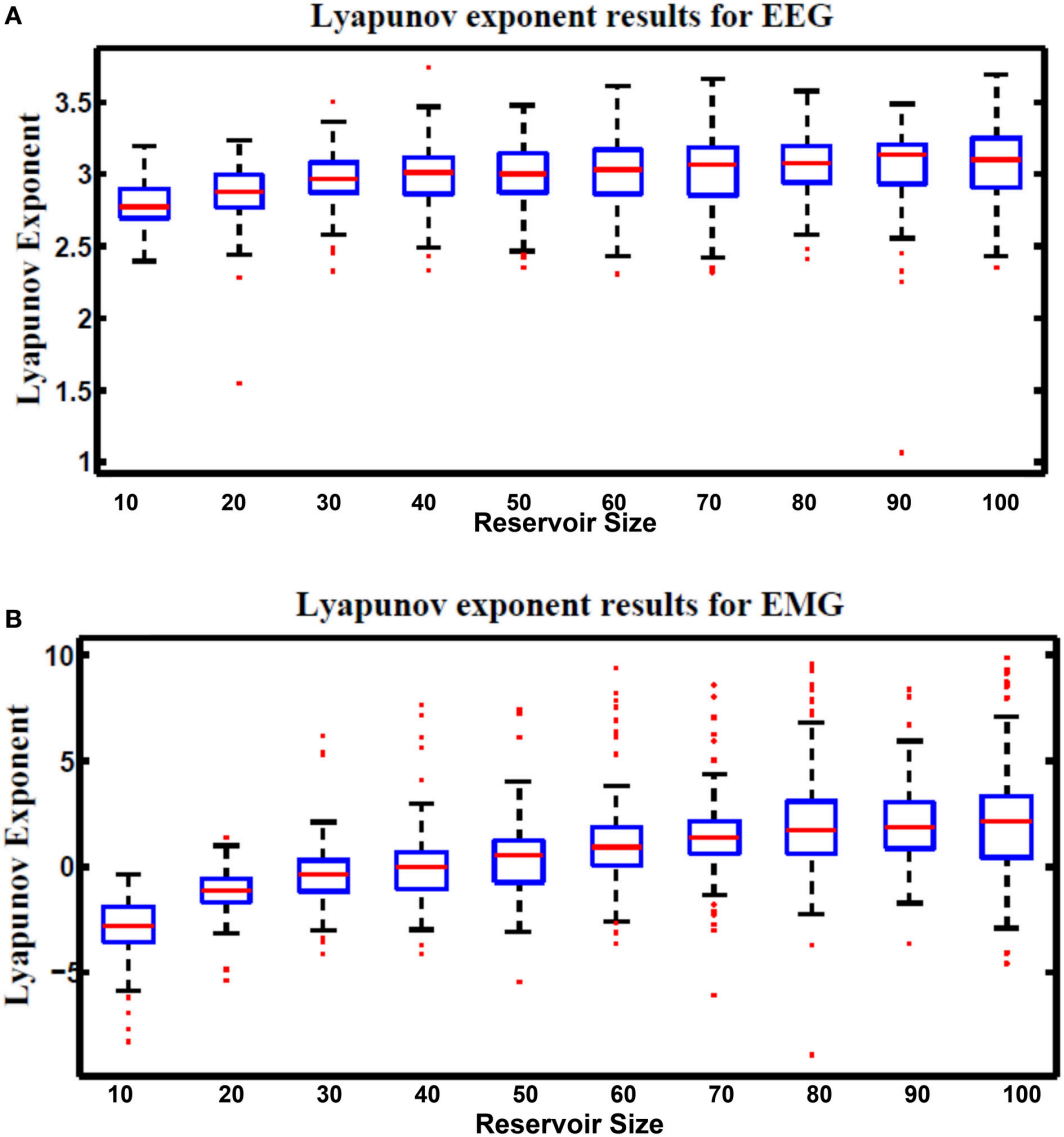
**Lyapunov's exponent results vs. different reservoir sizes for hybrid topology reservoir testing (A) EEG and (B) EMG signals**. The boxes represent the 25th and 75th percentiles of the measurements. The whiskers represent values outside the 25–75th percentiles.

Lyapunov's exponent is a measure of the chaos in the dynamic response of the reservoir. This metric was formulated in Gibbons (2010). Equation (29) is used to calculate Lyapunov's exponent value. Positive values of this metric represent the chaotic dynamic region while negative values represent the stable region. Since the optimal reservoir performance occurs at the edge of chaos (Natschläger, 2005), Lyapunov's exponent of zero is desirable.


(29) $$\lambda (t)=k\sum_{n=1}^{N}\ln\left(\frac{\left\|x_{j}(t)-x_{\hat{j}}(t)\right\|}{\left\|u_{j}(t)-u_{\hat{j}}(t)\right\|}\right)$$

where *N* is the total number of test cases used for the calculation, *k* is an undetermined scale factor that is varies based on the type and number of input vectors used in the computation. In this research *k* is chosen to be 1. *u*_*j*_(*t*) is an input to the reservoir at time step *t*. *u*_*ĵ*_(*t*) is the nearest neighbor to *u*_*j*_(*t*). *x*_*j*_(*t*) and *x*_*ĵ*_(*t*) are the reservoir response to *u*_*j*_(*t*) and *u*_*ĵ*_(*t*), respectively. Figure 14 shows Lyapunov's exponent results for the hybrid reservoir topology processing EEG and EMG signals. Results showed that the values of this metric are higher than zero for EEG signals while it showed that they are around zero for EMG signals. This means that the hybrid topology has more chaotic response to the EEG signals compared to the response of the EMG signals. It also showed that the size of the reservoir processing the EEG signals has low effect on the value of Lyapunov's exponent compared to the reservoir processing the EMG signals. As in kernel quality, there is variation in the values of Lyapunov's exponent and this is also attributed to the random weights of the input and reservoir synapses.

### 7.6. Power

**Figure 15:**
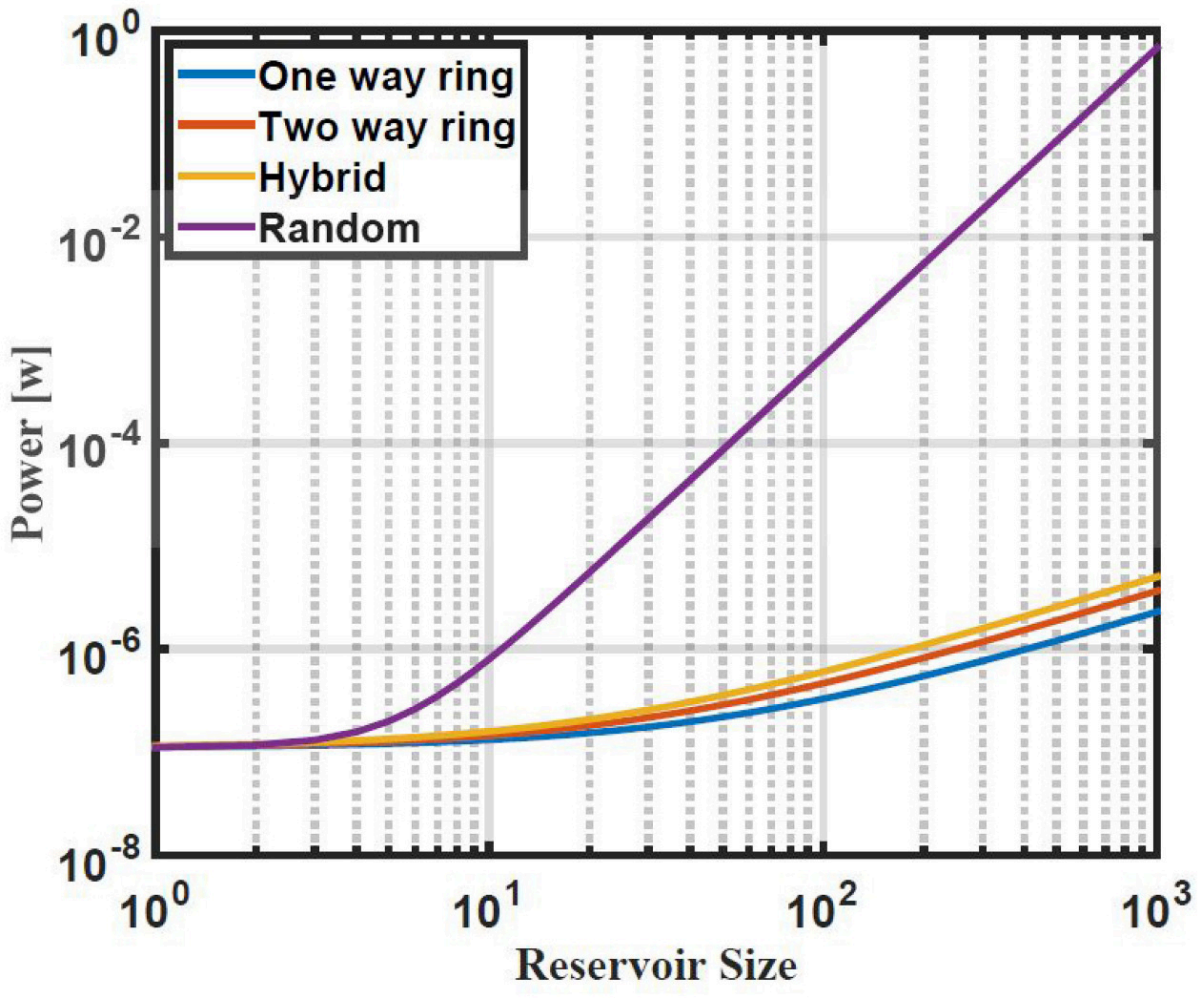
**Power consumption vs. reservoir size of four ESN topologies: one way ring, two way ring, hybrid, and random on log scale plot**. The one way ring topology has lower power consumption compared to the other topologies while the random topology has higher power consumption compared to the other topologies.

Power dissipation of four different reservoir topologies is quantitatively calculated using Equations (14), (23), and (28). The maximum current *I*_*max*_, VDD, and activity factor η used in the calculation are 1 nA, 0.55 V, and 0.5, respectively. Figure 15 shows the power dissipation of these reservoir topologies, one way ring, two way ring, hybrid, and random with 50% degree of connectivity over different reservoir sizes on a log scale plot. The power dissipation of the one way ring, two way ring, and hybrid topologies is in several micro watts. Results showed that the one way ring topology has lower power consumption compared to the two way ring and hybrid topologies. This is attributed to the small number of synapses that the one way ring topology has where each reservoir layer neuron has only one synapse. Results also showed that the relation between power dissipation of these three topologies and reservoir size is linear. This implies that there is no power dissipation overhead for increasing the size of the reservoir. The random topology has higher power dissipation (in several milliwatts) because it has a high number of synapses compared to the other topologies. It also showed that the power dissipation of the random topology is exponentially related to the size of the reservoir. For this reason the random topology is not desired for the hardware implementation.

Table 3 compares power consumption of both digital and mixed signal implementations of the 30 neurons hybrid reservoir. The performance of the digital realization is limited by the resources and logic blocks available on the FPGAs, with the Spartan-6LP-LX150T platform consuming the lowest power. As expected, using custom subthreshold circuits in the mixed-signal ESN design curtailed the power dissipation the most. These power savings, shown here, are within acceptable limits for power-constrained embedded platforms.

**Table 3:** **Power consumption of digital and mixed signal implementations of 30 neurons hybrid reservoir**.

Design	Power (mW)
Virtex5-LX110T	125.36
Virtex6-LX550TL	47.76
Spartan-6LP-LX150T	26.7
Mixed Signal	0.202*E* − 3

## 8. Conclusions

This research underpins that a scalable neuromemristive ESN architecture for power constrained applications is feasible. A double twisted toroidal ESN architecture with multichannel links is shown to achieve a classification accuracy of 90 and 84% for epileptic seizure detection and prosthetic finger control, respectively. The quality of the reservoir in the ESN is analyzed using kernel quality and Lyapunov's exponent metrics. Hardware realization of the ESN architecture was studied in two parts, a digital implementation and a mixed-mode design. Employing mismatches in transistors threshold voltages to design subthreshold bipolar synapses, yielded a good random distribution of weights in the ESN input and reservoir layer. Further, the power profile of the mixed-signal design is low for all the topologies due to the subthreshold NMS primitive circuits. Overall, the proposed architecture is generic and can also be validated for other biosignal processing applications. In the future, there is a need to develop metrics that meet multiple target criteria for the hardware RC architectures.

## Author contributions

All authors listed, have made substantial, direct and intellectual contribution to the work, and approved it for publication.

### Conflict of interest statement

The authors declare that the research was conducted in the absence of any commercial or financial relationships that could be construed as a potential conflict of interest.

## Footnotes

^1^The term neuron is used to refer to a node or cell in the ESN. The term node is used to refer to an electrical node or a node in the network architecture.
